# Unraveling the Molecular Puzzle: Exploring Gene Networks across Diverse EMT Status of Cell Lines

**DOI:** 10.3390/ijms241612784

**Published:** 2023-08-14

**Authors:** Heewon Park

**Affiliations:** School of Mathematics, Statistics and Data Science, Sungshin Women’s University, Seoul 02844, Republic of Korea; hwpark@sungshin.ac.kr

**Keywords:** gene regulatory network, explainable AI, epithelial–mesenchymal transition, precision medicine

## Abstract

Understanding complex disease mechanisms requires a comprehensive understanding of the gene regulatory networks, as complex diseases are often characterized by the dysregulation and dysfunction of molecular networks, rather than abnormalities in single genes. Specifically, the exploration of cell line-specific gene networks can provide essential clues for precision medicine, as this methodology can uncover molecular interplays specific to particular cell line statuses, such as drug sensitivity, cancer progression, etc. In this article, we provide a comprehensive review of computational strategies for cell line-specific gene network analysis: (1) cell line-specific gene regulatory network estimation and analysis of gene networks under varying epithelial–mesenchymal transition (EMT) statuses of cell lines; and (2) an explainable artificial intelligence approach for interpreting the estimated massive multiple EMT-status-specific gene networks. The objective of this review is to help readers grasp the concept of computational network biology, which holds significant implications for precision medicine by offering crucial clues.

## 1. Introduction

Recently, heterogeneous gene regulatory networks, which are regulatory interactions between genes controlling specific cell functions, have garnered a large amount of attention in various fields of research to understand disease mechanisms arising from complex molecular networks. To estimate gene regulatory networks, various computational methodologies have been developed. Furthermore, the effectiveness of these gene networks has been validated in various works, e.g., drug combinations identification, cancer prediction, etc. [1,2,3]. Although numerous studies on gene regulatory networks have been widely conducted, most of the existing studies have been based on averaged gene networks across all cell lines. Thus, we cannot reveal cell line (or patient)-specific gene regulatory networks that contain crucial clues for precision medicine.

In this article, we review computational strategies for cell line characteristic-specific gene network analysis. We first review machine learning approaches for sample-specific gene network estimation. We then focus on epithelial–mesenchymal transition (EMT), which is a biological phenomenon wherein epithelial cells undergo a transformation, leading to the loss of their characteristic properties and the acquisition of mesenchymal characteristics. The EMT plays key roles in cancer invasion, metastasis, and resistance to chemotherapy. Thus, uncovering the EMT mechanism is crucial for developing effective strategies to target cancer metastasis and enhance therapeutic efficacy. To understand the mechanism of transformation from epithelial cells to mesenchymal states and the related markers, we consider gene networks under varying EMT statuses of cell lines and review a study on uncovering system changes in gene networks under varying EMT statuses of cell lines [4]. We then turn our focus to the drawbacks in the studies involving the analysis of cell line characteristic-specific gene networks, specifically the interpretation of the massive multiple networks. The cell line-specific gene network analysis provides hundreds of networks for hundreds of cell lines, where a network is given in matrix form consisting of more than 10,000 rows with more than 1000 columns. Thus, the task of interpreting the massive multiple networks remains a big challenge. However, the inference of gene regulatory networks is not the final result; rather, these networks are intended to help solve biological and biomedical problems by effectively interpreting the estimated gene networks [5]. Gene regulatory networks play a crucial role in comprehending the maintenance, establishment, and disruption of cellular identity in diseases [6]. The gene networks facilitate our understanding of the molecular mechanisms governing organisms and reveal the fundamental principles governing a wide array of biological processes and reactions in organisms [7]. Furthermore, extensive evidence shows that epithelial–mesenchymal transition (EMT) is regulated by numerous transcription factors and signaling pathways, and gene regulatory networks play a critical role in controlling EMT programs during both developmental processes and disease [8,9].

In the second part of this article, we review the explainable artificial intelligence (XAI) approach, TRIP, which is used to interpret large-scale gene regulatory networks, enabling researchers to unravel complex biological processes and gain a deeper understanding of disease mechanisms [10]. We also review the application results of XAI in interpreting the estimated EMT status-specific gene networks [11]. TRIP was applied to gene networks estimated a decade ago and EMT markers were uncovered. Interestingly, some of the genes identified in this analysis were also identified as EMT markers in a previous study on EMT network analysis [4]. This implies that the dataset from 10 years ago contained knowledge of the EMT markers and their corresponding EMT-related mechanisms, which have subsequently been discovered in the last decade. Due to the lack of computational strategies for comprehensive analysis and interpretation of the massive gene regulatory networks, we were unable to uncover the EMT markers and their EMT-related mechanisms 10 years ago. Based on these reviews, it is expected that the incorporation of XAI will bring forth new possibilities in computational network biology, and may lead to a more effective understanding of complex disease mechanisms.

The remainder of this paper is organized as follows. In Section 2, we review the computational strategies for cell line-specific gene network analysis. The application results of the strategy for estimating cell line-specific gene networks under varying EMT statuses of cell lines are reviewed in Section 2. Then, we review the explainable artificial intelligence approach for interpreting the massive multiple networks in Section 3. The conclusions are provided in the Discussion section.

## 2. Investigating System Changes in Epithelial–Mesenchymal Transition through Personalized Gene Network Analysis

### 2.1. Computational Strategies

The expression levels of *p* regulator genes are given as $X={\left(x_{1},...,x_{n}\right)}^{T}\in R^{n\times p}$, where $x_{i}=\left(x_{i1},...,x_{ip}\right)$, which control target gene transcription $y_{\ell }\in R^{n}$ for *n* cell lines, ℓ=1,...,q. The gene regulatory network can be described by the following linear regression framework,

(1) $$\begin{array}{c} y_{i\ell }=\beta_{\ell }^{T}x_{i}+\epsilon_{i\ell },\;i=1,...,n,\;\ell =1,...,q, \end{array}$$

where $\beta_{\ell }={\left(\beta_{\ell 1},...,\beta_{\ell p}\right)}^{T}$ is the regression coefficient vector that represents the effect of *p* regulator genes on the $\ell^{th}$ target gene, and $\epsilon_{i\ell }$ is a random error vector.

The $L_{1}$-type regularization methods have been used to estimate gene regulatory networks, e.g., elastic net [12],

(2) $$\begin{array}{c} {\hat{\beta }}_{\ell }=\underset{\beta_{\ell }}{\arg\;\min}\left\{\frac{1}{2}\sum_{i=1}^{n}{\left(y_{i\ell }-\beta_{\ell }^{T}x_{i}\right)}^{2}+P_{\delta \lambda }\left(\beta_{\ell }\right)\right\}, \end{array}$$

where

$$\begin{array}{c} P_{\delta \lambda }\left(\beta_{\ell }\right)=\lambda \sum_{j=1}^{p}[\frac{1}{2}\left(1-\delta \right)\beta_{\ell j}^{2}+\delta |\beta_{\ell j}\left|\right] \end{array}$$

where λ>0 is a regularization parameter controlling the degree of shrinkage applied to $\beta_{\ell }$, and 0≤δ≤1 is a mixing parameter between the ridge [13] and lasso [14] penalties. Although the $L_{1}$-type regularization methodologies successfully estimate gene regulatory networks, the methods provide an averaged gene network that provides an average representation across various cell lines.

**Figure 1:**
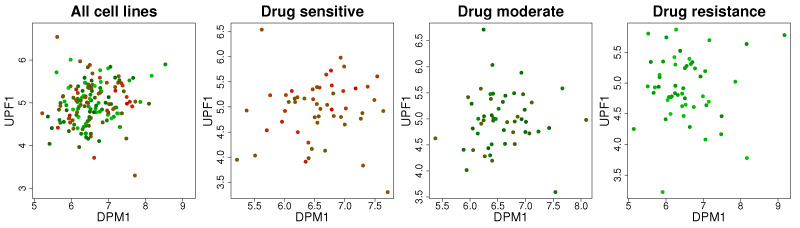
Correlation between two genes (i.e., UPF1 and MPM1) under varying conditions of cell line characteristics (i.e., all cell lines, anti-cancer drug-sensitive, -moderate, and -resistant cell lines).

However, molecular interplays exhibit diverse structures depending on the characteristics of cell lines. Figure 1 shows the correlation between two genes (i.e., UPF1 and DPM1) in cell lines corresponding to low (drug-sensitive), middle (drug-moderate), and high (drug-resistant) IC50 values of an anti-cancer drug (i.e., capecitabine). As shown in Figure 1, the two genes show a positive correlation in the drug-sensitive cell lines, whereas a negative correlation is seen in the drug resistance cell lines. Furthermore, the positive and negative correlation patterns between two genes cannot be discerned without considering the characteristics of cell lines (i.e., all cell lines). This implies that the characteristics of cell lines should be considered when estimating gene regulatory networks to extract crucial information for precision medicine. That is, cell line-specific gene regulatory networks are essential to accurately uncover the gene regulatory system for the specific characteristics of each cell line. RNA expression data were obtained from the CCLE dataset, and drug sensitivity data were downloaded from the Genomics of Drug Sensitivity in Cancer Project.

#### 2.1.1. NetworkProfiler

Shimamura et al. [4] developed a computational strategy to infer cell line-specific gene networks based on the following varying coefficient [15]

(3) $$\begin{array}{c} y_{i\ell }=\beta_{\ell }^{T}\left(m_{\alpha }\right)x_{i}+\varepsilon_{i\ell }, \end{array}$$

where $\beta_{\ell }\left(m_{\alpha }\right)$ is the regression coefficient vector that describes the effect of *p* regulator genes on the $\ell^{th}$ target gene in the $\alpha^{th}$ cell line. The $\alpha^{th}$ cell line has $m_{\alpha }$ as a cancer-related characteristic, where the characteristic of cell lines is referred to as a modulator *M* (e.g., EMT status of cell lines). To estimate the varying coefficient $\beta_{\ell }\left(m_{\alpha }\right)$, Shimamura et al. [4] developed the kernel-based L1-type regularization method as follows,

(4) $$\begin{array}{c} {\hat{\beta }}_{\ell }\left(m_{\alpha }\right)=\underset{\beta_{\ell }\left(m_{\alpha }\right)}{\arg\;\min}\left\{\frac{1}{2}\sum_{i=1}^{n}{\left\{y_{i\ell }-\beta_{\ell }^{T}\left(m_{\alpha }\right)x_{i\ell }\right\}}^{2}G\left(m_{i}-\;m_{\alpha }|b_{\ell }\right)+P\left\{\beta_{\ell }\left(m_{\alpha }\right)\right\}\right\} \end{array}$$

where $P\{\beta_{\ell }\left(m_{\alpha }\right)\}$ is the recursive elastic net penalty,

(5) $$\begin{array}{c} P\left\{\beta_{\ell }\left(m_{\alpha }\right)\right\}=\lambda_{\ell \alpha }\sum_{j=1}^{p}[\frac{1}{2}\left(1-\delta_{\ell \alpha }\right)\beta_{\ell j\alpha }^{2}+\delta_{\ell \alpha }w_{\ell j\alpha }|\beta_{\ell j\alpha }\left|\right], \end{array}$$

and

(6) $$\begin{array}{c} G\left(m_{i}-m_{\alpha }|b_{\ell }\right)=\exp\left\{\frac{-{\left(m_{i}\;-\;m_{\alpha }\right)}^{2}}{b_{\ell }}\right\}, \end{array}$$

is the Gaussian kernel function used for grouping cell lines according to their cancer-related characteristics (i.e., modulator $m_{i}$ for i=1,...,n). In other words, the Gaussian kernel function is employed to quantify the similarity between the characteristics of cell lines and determine the weights that control the influence of samples in estimating the gene network for the $\alpha^{th}$ cell line. Thus, we can estimate $\beta_{\ell }\left(m_{\alpha }\right)$ based only on cell lines having similar characteristics $m_{i}$ to those of the target cell line $m_{\alpha }$. This implies that NetworkProfiler has the ability to identify specific molecular interactions for cancer-related statuses of cell lines (e.g., EMT status, drug sensitivity, cancer progression-specific gene regulatory networks). In practice, genomic datasets frequently contain outliers originating from diverse sources, such as experimental errors, coding errors, and other factors. However, $L_{1}$-type regularization methods and NetworkProfiler suffer from outliers because the methods are based on the least-squares loss function. Thus, the network estimation and edge selection procedures are disturbed in the presence of outliers. In short, we cannot effectively perform personalized gene network analysis. In the subsequent subsection, we review robust personalized gene network analysis.

#### 2.1.2. Robust NetworkProfiler

In practice, clinical and genomic alteration datasets typically encompass outliers arising from multiple sources (e.g., experimental errors, reporting or labeling errors, etc.). The genomic dataset consists of a substantial number of features (e.g., genes) and a limited number of samples (e.g., cell lines). This type of data is called high-dimensional data. Identifying and managing outliers in a high-dimensional genomic dataset are crucial and challenging tasks. To effectively detect outliers in a high-dimensional genomic dataset, Park et al. [16] considered the following robust Mahalanobis distance computed in the robust principal component space,

(7) $$\begin{array}{c} R.MD_{i}^{r.pc}=\sqrt{{\left(z_{i}^{R}-T^{r.pc}\right)}^{T}{\left(C^{r.pc}\right)}^{-1}\left(z_{i}^{R}-T^{r.pc}\right)}, \end{array}$$

where $T^{r.pc}$ and $C^{r.pc}$ are the robust mean and covariance matrices, respectively, estimated using the minimum volume ellipsoid, and $Z^{R}={\left(z_{1}^{R},...,z_{n}^{R}\right)}^{T}$ is a κ-dimensional matrix of the robust principal components. By using the robust Mahalanobis distance, Park et al. [16] developed the following weight to control outliers in high-dimensional genomic data,

(8) $$\begin{array}{c} R_{i}^{\kappa }=\frac{\min(\sqrt{k/R.MD_{i}^{r.pc}},1)}{\sum_{i=1}^{n}\min(\sqrt{k/R.MD_{i}^{r.pc}},1)}, \end{array}$$

where $k=\chi^{2}\left(\mathrm{df}=\kappa \right)$ is the 95% quantile of the $\chi^{2}\left(\mathrm{df}=\kappa \right)$ distribution [17]. Park et al. [16] incorporated the weight into the kernel-based $L_{1}$-type regularization method as follows

(9) $$\begin{array}{c} {\hat{\beta }}_{\ell }\left(m_{\alpha }\right)=\underset{\beta_{\ell }\left(m_{\alpha }\right)}{\arg\;\min}\left\{\frac{1}{2}\sum_{i=1}^{n}R_{i}^{\kappa }{\left\{y_{i\ell }-\beta_{\ell }^{T}\left(m_{\alpha }\right)x_{i\ell }\right\}}^{2}G\left(m_{i}-m_{\alpha }|b_{\ell }\right)+P\left\{\beta_{\ell }^{T}\left(m_{\alpha }\right)\right\}\right\}. \end{array}$$

The robust NetworkProfiler detects data points as outliers if their $R.MD_{i}^{r.pc}$ is greater than the 95th percentile of the $\chi^{2}\left(df=\kappa \right)$ distribution, and then reduces the effect of the detected outliers by applying the weight $R_{i}^{\kappa }$ to the network estimation. Due to its robust nature, NetworkProfiler can effectively estimate personalized gene networks, even in the presence of outliers.

### 2.2. Uncovering Changes in Gene Regulatory Networks in the Epithelial–Mesenchymal Transition

We review the application results of NetworkProfiler for gene network analysis under varying EMT statuses of cell lines. Shimamura et al. [4] applied NetworkProfiler to reveal system changes under varying EMT statuses of cell lines. EMT status-specific gene networks were estimated using the EMT modulator that describes the EMT statuses of cell lines, defined using the module discovery method [18]. This method is based on 50 genes labeled as EMT-related genes (i.e., EMT-UP, EMT-DN, JECHLIN-GER-EMT-UP, and JECHLIN-GER-EMT-DN) in the Molecular Signatures Database v2.5, where low and high values of the EMT modulator represent the epithelial- and mesenchymal-like cell lines, respectively. The expression profiles of 13,508 genes in 762 cell lines were obtained from the Sanger Cell Line Project (http://bonsai.hgc.jp/~shima/NetworkProfiler/, accessed on 25 June 2023). The gene networks were estimated with 13,508 target genes and 1732 regulator genes, consisting of 47 nuclear receptors, 1183 transcription factors, and 502 human miRNA. For the 762 modulator values describing the EMT statuses of 762 cell lines, 762 gene regulatory networks between the 13,508 target and 1732 regulator genes were estimated.

To reveal system changes in the context of EMT, Shimamura et al. [4] focused on the well-known EMT marker, E-cadherin, because the loss of the cell adhesion molecule E-cadherin is a biomarker of EMT. Then, they identified candidate regulators of E-cadherin based on the following regulatory effect of the $j^{th}$ regulator on the $l^{th}$ target gene (i.e., E-cadherin) at the $\alpha^{th}$ cell line,

(10) $$\begin{array}{c} {\mathrm{RE}}_{j\ell \alpha }=\sum_{s\in \pi_{j\ell \alpha }}{\hat{\beta }}_{s}^{\left(j\to \ell \right)}\left(m_{\alpha }\right)\cdot x_{\alpha j}. \end{array}$$

where $\pi_{j\ell \alpha }$ is the set of all possible paths from $x_{j}$ (i.e., regulators) to $y_{\ell }$ (i.e., E-cadherin), and ${\hat{\beta }}^{\left(j\to \ell \right)}\left(m_{\alpha }\right)$ is the product of the estimated coefficients on the $\ell^{th}$ path in $\pi_{j\ell \alpha }$, where the length of the path from $x_{j}$ to $y_{\ell }$ was regarded as 1 or 2. In other words, they considered the parent and grandparent genes as potential regulatory factors. Then, the following regulatory effect change according to the EMT status of cell lines was computed,

(11) $$\begin{array}{c} {\mathrm{REC}}_{j\ell }=\max\left\{{\mathrm{RE}}_{jl\alpha };\alpha =1,...,n\right\}-\min\left\{{\mathrm{RE}}_{j\ell \alpha };\alpha =1,...,n\right\}, \end{array}$$

to measure how the EMT status affects the regulatory effect of the regulators on E-cadherin. The highest ranked 25 genes corresponding the highest REC values were extracted as candidate regulators of E-cadherin: IRF6 (-), miR-141 ([19]), GRHL2 (-), ZEB1 ([20]), LSR (-), miR-200b ([19]), KLF4 ([21]), OVOL2 (-), miR-200a ([19]), FOXA2 ([22]), TCF4 ([23]), ELF3 (-), SNF17 (-), MYB (-), KLF5 (-), miR-192 ([24]), FOXA1 ([23]), SNF165 (-), NKX2-1 (-), HNF1B (-), TFE3 (-), ZEB2 ([25]), TRIM29 (-), SNAI2 ([26]), where reference numbers in brackets indicate the previous studies on the regulatory mechanisms of the genes related to E-cadherin.

Among the 25 candidate regulators identified, about half of the genes had well-established evidence supporting their regulatory roles in E-cadherin, whereas the mechanisms of the remaining genes were yet to be revealed. NetworkProfiler was employed to predict the mechanistic interpretations of the E-cadherin-related regulatory system as follows:

- The expression of miR-141 had a strong positive effect on the expression of E-cadherin in epithelial-like cells, whereas this effect decreased as the transition from epithelial- to mesenchymal-like cell lines occurred.
- The expression of ZEB1 had a weak negative effect on the expression of E-cadherin in epithelial-like cells, whereas this effect increased as the transition from epithelial- to mesenchymal-like cell lines occurred.
- miR-141 and ZEB1 had a strong negative effect on each other only in epithelial-like cells.

The findings suggest the existence of an adverse feedback loop between miR-141 and ZEB1 in epithelial-like cells, and this interaction had been previously revealed in [27].

- As the transition from epithelial-like cells to mesenchymal-like cells occurred, the expression levels of miR-141 and E-cadherin decreased, whereas the expression level of ZEB1 increased.

Shimamura et al. [4] suggested, based on the aforementioned results, that the inhibition of miR-141 in mesenchymal-like cells disrupts the adverse feedback loop between miR-141 and ZEB1, consequently resulting in decreased expression of E-cadherin due to the increased expression of ZEB1.

### 2.3. Limitations of Current Personalized Gene Network Analysis

Personalized gene network analysis (e.g., EMT status-specific gene network) generates massive multiple networks, where each network is represented in matrix form with 1732 columns and 13,508 rows. This indicates that the interpretation of the analysis of EMT status-specific gene networks involves the examination of 762 extensive matrices, with each matrix corresponding to a distinct cell line.

Although NetworkProfiler enables us to explore changes in the system under different EMT status conditions, the existing study focused only on the well-known EMT marker, E-cadherin, and then interpreted the results based on the neighboring genes of E-cadherin. This approach was taken as the comprehensive analysis and interpretation of the extensive multiple networks were not feasible. However, the narrow interpretation is insufficient to understand the complex mechanism of disease. To effectively advance precision medicine, a comprehensive interpretation of the massive multiple gene networks is essential.

## 3. Explainable Artificial Intelligence (XAI) for Comprehensive Gene Network Analysis

In this section, we review the explainable artificial intelligence approach, known as Tensor Reconstruction-based Interpretable Prediction (TRIP), for the analysis of massive multiple networks [10]. In recent years, artificial intelligence has garnered considerable attention in various fields of research, statistics, computer science, biomedical, etc. Although AI approaches have shown significant success in terms of prediction or classification accuracy, the methods frequently encounter the black-box problem, wherein the decision-making process of AI machines cannot be explained due to the highly intricate nature of the deep learning model’s decision rules. The utilization of AI in the medical field is limited because a basis for explanation is required. To address the black-box problem, Maruhashi et al. [10] developed an XAI strategy called TRIP, which is a deep learning approach for tensor decomposition. It aims to find a subspace of the data from multiple networks that minimizes prediction error while retaining as much of the data information as possible. TRIP estimates a human-readable low-dimensional subspace and performs predictions based on the estimated subspace. Thus, we can effectively interpret and understand the analysis of massive multiple networks because the decision boundaries of a model can be efficiently visualized in a lower human-readable dimension, even though the model is learned through a deep learning approach. We briefly review the mathematical formula of TRIP in the following subsection.

### 3.1. Method: Tensor Reconstruction-Based Interpretable Prediction (TRIP)

The gene network connecting target genes with regulator genes can be regarded as a second-order tensor. Assume a *K*-mode tensor X with size $I_{1}\times \cdots \times I_{K}$. TRIP estimates a projection matrix $C^{\left(k\right)}\in R^{I_{k}\times J_{k}}$ for X and projects X onto a lower-dimensional subspace using the estimated $C^{\left(k\right)}$, i.e.,

(12) $$\begin{array}{c} {\bar{X}}_{i}=X_{i}\prod_{k}\times_{k}C^{\left(k\right)}. \end{array}$$

Then, the projected tensor ${\bar{X}}_{i}$ is used as the input for the prediction or classification model, as follows

(13) $$\begin{array}{c} {\hat{y}}_{i}=f\left({\bar{X}}_{i},\theta \right)\;\mathrm{for}\;i=1,...,n. \end{array}$$

This implies that the prediction or classification is conducted within the estimated human-readable low-dimensional subspace. The projection-based prediction leads to more explainable and interpretable results in the analysis of multilayer gene networks because the complex massive multiple gene networks are effectively visualized within a human-readable dimensional subspace.

TRIP estimates the projection matrices $C^{\left(k\right)}$ and learns the deep learning-based prediction model simultaneously by minimizing not only the prediction error but also the subspace estimation error. The objective function of TRIP is given as

(14) $$\begin{array}{c} O_{T}=\frac{1}{n}\sum_{i=1}^{n}\{L\left(y_{i},{\hat{y}}_{i}\right)+\gamma ∥X_{i}-{\bar{X}}_{i}\prod_{k}\times_{k}C^{(k)T}{∥}_{2}^{2}\},\\ \mathrm{subject}\mathrm{to}\;C^{(k)T}C^{\left(k\right)}=I, \end{array}$$

where γ>0 is the tuning parameter for the projection error. The first term $L(y_{i},{\hat{y}}_{i})$ and the second term are the loss functions for predicting and estimating the projection matrices, respectively. As shown in the objective function (14), TRIP learns a model by minimizing these two loss functions simultaneously. In other words, the projection matrices $C^{\left(k\right)}$ are estimated to minimize errors in both projection and prediction. This implies that TRIP enables us to achieve effective prediction results while still retaining a significant amount of the original data variance within the estimated subspace. For details about TRIP, please refer to Maruhashi et al. [10]

### 3.2. Comprehensive Interpretation of the Massive Multiple Gene Networks across Varying EMT Statuses

**Figure 2:**
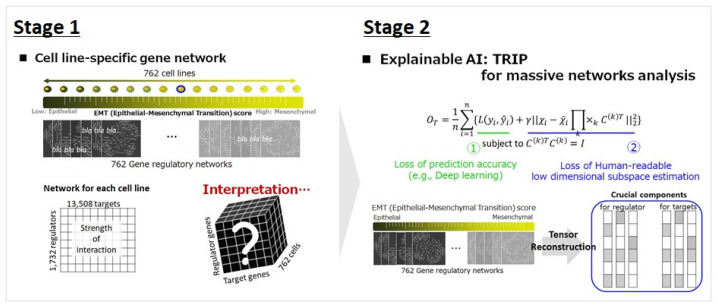
Overall framework of EMT network analysis: (1) Estimation of EMT status-specific gene networks for 762 cell lines. (2) Interpretation of the massive multiple gene networks using the explainable AI technique, TRIP. (3) Interpretation of the networks based on the first three crucial components of the regulator axis.

Park et al. [11] applied TRIP to estimate 762 gene regulatory networks across varying EMT statuses of cell lines. The gene network between 13,508 targets with 1762 regulators was regarded as a second-order tensor. TRIP first estimated the projection matrices $C^{\left(k\right)},k\;=\;1,2$ for the regulator and target genes axes and learned a 50×50 subspace of the 762 networks. Then, 50 crucial components that describe the importance of the target and regulator genes for EMT-modulator prediction were extracted. Park et al. [11] interpreted the results based on the crucial components of the subspace for regulator genes. Among the estimated 50 components, the first three components explained approximately 70% of the variability in the regulator genes within EMT-related gene networks (first component: 56%; second component: 8%; third component: 4%). Then, the interpretation of the EMT networks was conducted using the first three components. Figure 2 shows the overall framework of the EMT network analysis. In other words, EMT status-specific gene networks were estimated for 762 cell lines. To interpret the massive multiple gene networks, the explainable AI technique, TRIP, was applied. The interpretation of these networks was based on the first three crucial components of the regulator axis.

In order to achieve a more biologically reliable interpretation, Park et al. [11] combined the results obtained using TRIP with well-known EMT markers, i.e., ZEB1, ZEB2, SNAIL1, SNAIL2, and TWIST1 (EMT-TFs). The target networks of the five EMT-TFs were extracted separately for the high- and low-value regions of each component. The target genes (TG.EMT-TFs) of the EMT-TFs and the target genes of the TG.EMT-TFs were also extracted. For the networks in the high and low regions of each component, the binary adjacency matrices were computed. The 10 genes with the most significant differences in edge structure between the binary adjacency matrices for the high and low regions of each component were extracted. Table 1 shows the identified genes from the three components and their EMT-related evidence, where “◯” in the column “In NetworkProfiler” indicates that the genes were also identified as top-25-ranked regulators of E-cadherin in the previous study of EMT status-specific gene network [4].

Most of the EMT markers identified using TRIP have been supported by strong evidence as EMT markers, and their EMT-related mechanisms have been previously reported in multiple studies. This implies that TRIP has provided biologically reliable insights into the identification of EMT-related mechanisms. Out of the 17 genes identified, GRHL2, IRF6, LSR, and OVOL2 were also identified as EMT markers (i.e., regulators of E-cadherin) in the previous EMT network analysis using the NetworkProfiler [4]. The EMT-related mechanisms of genes such as GRHL2, IRF6, LSR, and OVOL2 were not known a decade ago. However, over the past decade, the EMT-related mechanisms of the genes have been uncovered. Interestingly, the EMT status-specific gene networks were estimated a decade ago based on data from that time, and TRIP was applied to the data to identify the EMT-related mechanisms. This implies that the data from 10 years ago already contained insights into EMT mechanisms that have since been revealed in the past decade. Because of a shortage of computational strategies for comprehensive analysis of the large-scale gene regulatory network, we were unable to reveal EMT-related mechanisms a decade ago (e.g., GRHL2, IRF6, OVOL2, LSR shown in Table 1). The application of the explainable AI technique, TRIP, allowed us to uncover the EMT-related mechanisms that have been unveiled over the past ten years in a single, unified analysis. Reviews on EMT gene network analysis indicate that the application of explainable AI could usher in a new era for computational network biology, potentially revolutionizing our understanding of intricate disease mechanisms.

## 4. Discussion

The aim of this review paper was to provide insights into the computational strategies for cell line-specific gene networks and the use of the explainable AI approach to overcome the bottleneck of existing black-box AI (i.e., interpreting the massive multiple gene networks). We also reviewed computational strategies for analyzing massive multiple gene networks. Although many studies have been conducted on gene network analysis, the existing studies were related to averaged gene networks for all cell lines (or patients), which cannot provide cell line-specific molecular characteristics. To address this issue, recently, computational strategies for cell line-specific gene network estimation have been developed. Cell line characteristic-specific gene network analysis enables us to uncover gene regulatory systems for specific characteristics of cell lines. The gene networks identified through this approach provide indispensable information that can significantly contribute to precision medicine. However, interpreting the massive multiple gene networks remains a challenge, mainly due to the extensive scale of the networks involved. Each interpretation subject entails hundreds of massive networks, comprising more than 10,000 rows and over 1000 columns. We reviewed the computational strategies for cell line-specific gene network estimation and the explainable AI approach for interpreting the estimated massive multiple gene regulatory networks. We also reviewed the analysis results of the gene regulatory system changes under varying EMT statuses of cell lines. Based on the reviews of two studies, it was found that the explainable AI approach, TRIP, has successfully unraveled the EMT-related mechanisms that have been discovered over the past ten years. This implies that the lack of computational strategies for the comprehensive analysis of large-scale gene networks has hindered the identification of the EMT-related mechanisms that have been revealed in the past ten years. We expect that explainable AI methods, such as TRIP, can offer insightful and interpretable solutions for understanding the complex dynamics of cancer network biology.

In this article, we have reviewed computational strategies for cell line-specific gene network analysis and their application for EMT status-specific gene networks. The Connectivity Map is one of the most powerful tools for identifying connections among small molecules sharing a mechanism of action, chemicals and physiological processes, and diseases and drugs [61]. It can be suggested that the use of a Connectivity Map with the reviewed strategies can lead to more comprehensive results, especially in the interpretation of drug-related characteristic-specific gene network analysis.

## Figures and Tables

**Table 1:** Identified novel candidate markers involved in EMT related mechanism [11].

Gene	Components	References	In NetworkProfiler
AFF1	3	-	-
ANKRD5	1	[28]	-
FOXF1	2	[29,30]	-
FOXF2	1	[31,32]	-
GLI3	2	[33,34]	-
GRHL2	1	[35,36,37,38]	◯
IFI16	2, 3	[39,40,41,42]	-
IRF6	3	[43,44]	◯
KANK2	3	-	-
LSR	2	[45,46,47,48]	◯
MAFB	3	-	-
OVOL2	2	[49,50,51,52]	◯
PCBD1	2	-	-
SOX13	2	[53]	-
TGFB1I1	2	[54,55,56,57]	-
TP63	2, 3	[58,59,60]	-
ZNF91	1	-	-

## Data Availability

The estimated networks can be downloaded from http://bonsai.hgc.jp/~shima/NetworkProfiler/, accessed on 25 June 2023.

## References

[1] Daoud M., Mayo M. (2019). A survey of neural network-based cancer prediction models from microarray data. Artif. Intell. Med..

[2] Cheng F., Kovacs I., Barabasi A. (2019). Network-based prediction of drug combinations. Nat. Commun..

[3] Fout A., Byrd J., Shariat B., Ben-Hur A. Protein interface prediction using graph convolutional networks. Proceedings of the NIPS’17: Proceedings of the 31st International Conference on Neural Information Processing Systems, Long Beach, CA, USA, 4–9 December 2017.

[4] Shimamura T., Imoto S., Shimada Y., Hosono Y., Niida A., Nagasaki M., Yamaguchi R., Takahashi T., Miyano S. (2011). A novel network profiling analysis reveals system changes in epithelial-mesenchymal transition. PLoS ONE.

[5] Emmert-Streib F., Dehmer M., Haibe-Kains B. (2014). Gene regulatory networks and their applications: Understanding biological and medical problems in terms of networks. Front. Cell Dev. Biol..

[6] Badia-I-Mompel P., Wessels L., Müller-Dott S., Trimbour R., Ramirez Flores R.O., Argelaguet R., Saez-Rodriguez J. (2023). Gene regulatory network inference in the era of single-cell multi-omics. Nat. Rev. Genet..

[7] Zhao M., He W., Tang J., Zou Q., Guo F. (2021). A comprehensive overview and critical evaluation of gene regulatory network inference technologies. Brief. Bioinform..

[8] Lavin D.P., Tiwari V.K. (2020). Unresolved Complexity in the Gene Regulatory Network Underlying EMT. Front. Oncol..

[9] Fazilaty H., Rago L., Kass Youssef K., Ocana O.H., Garcia-Asencio F., Arcas A., Galceran J., Nieto M.A. (2019). A gene regulatory network to control EMT programs in development and disease. Nat. Commun..

[10] Maruhashi K., Park H., Yamaguchi R., Miyano S. (2020). Linear Tensor Projection Revealing Nonlinearity. arXiv.

[11] Park H., Maruhashi K., Yamaguchi R., Imoto S., Miyano S. (2020). Global gene network exploration based on explainable artificial intelligence approach. PLoS ONE.

[12] Zou H., Hastie T. (2005). Regularization and variable selection via the elastic net. J. R. Stat. Soc. Ser. B.

[13] Hoerl A.E., Kennard R.W. (1970). Ridge regression: Biased estimation for nonorthogonal problems. Techonometrics.

[14] Tibshirani R. (1996). Regression shrinkage and selection via the lasso. J. R. Stat. Soc. Ser. B.

[15] Hastie T., Tibshirani R. (1993). Varying-Coefficient Models. J. R. Stat. Soc. Ser. B.

[16] Park H., Shimamura T., Miyano S., Imoto S. (2014). Robust prediction of anti-cancer drug sensitivity and sensitivity-specific biomarker. PLoS ONE.

[17] Khan J.A., Van Aelst S., Zamar R.H. (2007). Robust linear model selection based on least angle regression. J. Am. Stat. Assoc..

[18] Niida A., Smith A.D., Imoto S., Aburatani H., Zhang M.Q., Akiyama T. (2009). Gene set-based module discovery in the breast cancer transcriptome. BMC Bioinf..

[19] Gregory P.A., Bert A.G., Paterson E.L., Barry S.C., Tsykin A., Farshid G., Vadas M.A., Khew-Goodall Y., Goodall G.J. (2008). The miR-200 family and miR-205 regulate epithelial to mesenchymal transition by targeting ZEB1 and SIP1. Nat. Cell Biol..

[20] Comijn J., Berx G., Vermassen P., Verschueren K., van Grunsven L., Bruyneel E., Mareel M., Huylebroeck D., van Roy F. (2001). The two-handed E box binding zinc finger protein SIP1 downregulates Ecadherin and induces invasion. Mol. Cell.

[21] Yori J.L., Johnson E., Zhou G., Jain M.K., Keri R.A. (2010). Kruppel-like factor 4 inhibits epithelial-tomesenchymal transition through regulation of E-cadherin gene expression. J. Biol. Chem..

[22] Song Y., Washington M.K., Crawford H.C. (2010). Loss of FOXA1/2 is essential for the epithelialto-mesenchymal transition in pancreatic cancer. Cancer Res..

[23] Sobrado V.R., Moreno-Bueno G., Cubillo E., Holt L.J., Nieto M.A., Portillo F., Cano A. (2009). The class I bHLH factors E2-2A and E2-2B regulate EMT. J. Cell Sci..

[24] Kato M., Zhang J., Wang M., Lanting L., Yuan H., Rossi J.J., Natarajan R. (2007). MicroRNA-192 in diabetic kidney glomeruli and its function in TGF-beta-induced collagen expression via inhibition of E-box repressors. Proc. Natl. Acad. Sci. USA.

[25] Eger A., Aigner K., Sonderegger S., Dampier B., Oehler S., Schreiber M., Berx G., Cano A., Beug H., Foisner R. (2005). DeltaEF1 is a transcriptional repressor of E-cadherin and regulates epithelial plasticity in breast cancer cells. Oncogene.

[26] Hajra K.M., Chen D.Y., Fearon E.R. (2002). The SLUG zinc-finger protein represses E-cadherin in breast cancer. Cancer Res..

[27] Bracken C.P., Gregory P.A., Kolesnikoff N., Bert A.G., Wang J., Shannon M.F., Goodall G.J. (2008). A double-negative feedback loop between ZEB1-SIP1 and the microRNA-200 family regulates epithelial-mesenchymal transition. Cancer Res..

[28] Daniel J.G., Panizzi J.R. (2019). Spatiotemporal expression profile of embryonic and adult ankyrin repeat and EF-hand domain containing protein 1-encoding genes ankef1a and ankef1b in zebrafish. Gene Exp. Patterns..

[29] Wang S., Yan S., Zhu S., Zhao Y., Yan J., Xiao Z., Bi J., Qiu J., Zhang D., Hong Z. (2018). FOXF1 Induces Epithelial-Mesenchymal Transition in Colorectal Cancer Metastasis by Transcriptionally Activating SNAI1. Neoplasia.

[30] Wei H.J., Nickoloff J.A., Chen W.H., Liu H.Y., Lo W.C., Chang Y.T., Yang P.C., Wu C.W., Williams D.F., Gelovani J.G. (2014). FOXF1 mediates mesenchymal stem cell fusion-induced reprogramming of lung cancer cells. Oncotarget.

[31] Lo P.K. (2017). The controversial role of forkhead box F2 (FOXF2) transcription factor in breast cancer. PRAS Open..

[32] Cai J., Tian A.X., Wang Q.S., Kong P.Z., Du X., Li X.Q., Feng Y.M. (2015). FOXF2 suppresses the FOXC2-mediated epithelial-mesenchymal transition and multidrug resistance of basal-like breast cancer. Cancer Lett..

[33] Iwasaki H., Nakano K., Shinkai K., Kunisawa Y., Hirahashi M., Oda Y., Onishi H., Katano M. (2013). Hedgehog Gli3 activator signal augments tumorigenicity of colorectal cancer via upregulation of adherence-related genes. Cancer Sci..

[34] Rodrigues M.F.S.D., Miguita L., De Andrade N.P., Heguedusch D., Rodini C.O. (2018). GLI3 knockdown decreases stemness, cell proliferation and invasion in oral squamous cell carcinoma. Int. J. Oncol..

[35] Chung V.Y., Tan T.Z., Tan M., Wong M.K., Kuay K.T., Yang Z., Ye J., Muller J., Koh C.M., Guccione E. (2016). GRHL2-miR-200-ZEB1 maintains the epithelial status of ovarian cancer through transcriptional regulation and histone modification. Sci. Rep..

[36] Xiang J., Fu X., Ran W., Wang Z. (2017). Grhl2 reduces invasion and migration through inhibition of TGF*β*-induced EMT in gastric cancer. Oncogenesis.

[37] Cieply B., Farris J., Denvir J., Ford H.L., Frisch S.M. (2013). Epithelial-mesenchymal transition and tumor suppression are controlled by a reciprocal feedback loop between ZEB1 and Grainyhead-like-2. Cancer Res..

[38] Mooney S.M., Talebian V., Jolly M.K., Jia D., Gromala M., Levine H., McConkey B.J. (2017). The GRHL2/ZEB Feedback Loop-A Key Axis in the Regulation of EMT in Breast Cancer. J. Cell Biochem..

[39] Alimirah F., Chen J., Davis F.J., Choubey D. (2007). IFI16 in human prostate cancer. Mol. Cancer Res..

[40] Lin W., Zhao Z., Ni Z., Zhao Y., Du W., Chen S. (2017). IFI16 restoration in hepatocellular carcinoma induces tumour inhibition via activation of p53 signals and inflammasome. Cell Prolif..

[41] Unterholzner L., Keating S.E., Baran M., Horan K.A., Jensen S.B., Sharma S., Sirois C.M., Jin T., Latz E., Xiao T.S. (2010). IFI16 is an innate immune sensor for intracellular DNA. Nat. Immunol..

[42] Roy A., Ghosh A., Kumar B., Chandran B. (2019). IFI16, a nuclear innate immune DNA sensor, mediates epigenetic silencing of herpesvirus genomes by its association with H3K9 methyltransferases SUV39H1 and GLP. eLife.

[43] Ke C.Y., Xiao W.L., Chen C.M., Lo L.J., Wong F.H. (2015). IRF6 is the mediator of TGF*β*3 during regulation of the epithelial mesenchymal transition and palatal fusion. Sci. Rep..

[44] Li D., Cheng P., Wang J., Qiu X., Zhang X. (2019). IRF6 Is Directly Regulated by ZEB1 and ELF3, and Predicts a Favorable Prognosis in Gastric Cancer. Front. Oncol..

[45] Shimada H., Abe S., Kohno T., Satohisa S., Konno Y. (2017). Loss of tricellular tight junction protein LSR promotes cell invasion and migration via upregulation of TEAD1/AREG in human endometrial cancer. Sci. Rep..

[46] Parsana P., Amend S.R., Hernandez J., Pienta K.J., Battle A. (2017). Identifying global expression patterns and key regulators in epithelial to mesenchymal transition through multi-study integration. BMC Cancer..

[47] Reaves D.K., Fagan-Solis K.D., Dunphy K., Oliver S.D., Scott D.W., Fleming J.M. (2014). The role of lipolysis stimulated lipoprotein receptor in breast cancer and directing breast cancer cell behavior. PLoS ONE.

[48] Takano K., Kakuki T., Obata K., Nomura K., Miyata R., Kondo A., Kurose M., Kakiuchi A., Kaneko Y., Kohno T. (2016). The Behavior and Role of Lipolysis-stimulated Lipoprotein Receptor, a Component of Tricellular Tight Junctions, in Head and Neck Squamous Cell Carcinomas. Anticancer Res..

[49] Liu J., Wu Q., Wang Y., Wei Y., Wu H., Duan L., Zhang Q., Wu Y. (2018). Ovol2 induces mesenchymal-epithelial transition via targeting ZEB1 in osteosarcoma. Onco Targets Ther..

[50] Nilsson G., Kannius-Janson M. (2016). Forkhead Box F1 promotes breast cancer cell migration by upregulating lysyl oxidase and suppressing Smad2/3 signaling. BMC Cancer.

[51] Roca H., Hernandez J., Weidner S., McEachin R.C., Fuller D. (2013). Transcription factors OVOL1 and OVOL2 induce the mesenchymal to epithelial transition in human cancer. PLoS ONE.

[52] Hong T., Watanabe K., Ta C.H., Villarreal-Ponce A., Nie Q., Dai X. (2015). An Ovol2-Zeb1 Mutual Inhibitory Circuit Governs Bidirectional and Multi-step Transition between Epithelial and Mesenchymal States. PLoS Comput. Biol..

[53] Zhang Y., Liao Y., Chen C., Sun W., Sun X., Liu Y., Xu E., Lai M., Zhang H. (2020). p38 regulated FOXC1 stability is required for colorectal cancer metastasis. J. Pathol..

[54] Chandhoke A.S., Karve K., Dadakhujaev S., Netherton S., Deng L., Bonni S. (2016). The ubiquitin ligase Smurf2 suppresses TGF*β*-induced epithelial-mesenchymal transition in a sumoylation-regulated manner. Cell Death Differ..

[55] Huang Y., Tong J., He F., Yu X., Fan L., Hu J., Tan J., Chen Z. (2014). miR-141 regulates TGF-*β*1-induced epithelial-mesenchymal transition through repression of HIPK2 expression in renal tubular epithelial cells. Int. J. Mol. Med..

[56] Moustakas A., Heldin C.H. (2016). Mechanisms of TGF*β* Induced Epithelial-Mesenchymal Transition. J. Clin. Med..

[57] Saito R.A., Watabe T., Horiguchi K., Kohyama T., Saitoh M., Nagase T., Miyazono K. (2009). Thyroid transcription factor-1 inhibits transforming growth factor-beta-mediated epithelial-to-mesenchymal transition in lung adenocarcinoma cells. Cancer Res..

[58] Zhang Y., Yan W., Chen X. (2014). P63 regulates tubular formation via epithelial-to-mesenchymal transition. Oncogene.

[59] Olsen J.R., Oyan A.M., Rostad K., Hellem M.R., Liu J., Li L., Micklem D.R., Haugen H., Lorens J.B., Rotter V. (2013). p63 attenuates epithelial to mesenchymal potential in an experimental prostate cell model. PLoS ONE.

[60] Lindsay J., McDade S.S., Pickard A., McCloskey K.D., McCance D.J. (2011). Role of DeltaNp63gamma in epithelial to mesenchymal transition. J. Biol. Chem..

[61] Lamb J., Crawford E.D., Peck D., Modell J.W., Blat I.C., Wrobel M.J., Lerner J., Brunet J.P., Subramanian A., Ross K.N. (2006). The Connectivity Map: Using gene-expression signatures to connect small molecules, genes, and disease. Science.

